# Secondary malignancies after partial versus whole breast irradiation: a systematic review and meta-analysis

**DOI:** 10.18632/oncotarget.12442

**Published:** 2016-10-04

**Authors:** Xin-Bin Pan, Shi-Ting Huang, Yan-Ming Jiang, Jia-Lin Ma, Xiao-Dong Zhu

**Affiliations:** ^1^ Department of Radiation Oncology, Cancer Hospital of Guangxi Medical University, Nanning, Guangxi 530021, P.R. China

**Keywords:** breast cancer, breast-conserving therapy, partial breast irradiation, whole breast irradiation, secondary malignancies

## Abstract

Secondary malignancies are a common complication for patients receiving radiotherapy. Here, we compared rates of secondary malignancies after partial breast irradiation (PBI) and whole breast irradiation (WBI). The MEDLINE, EMBASE, and the Cochrane Library databases were systematically searched to identify relevant randomized clinical trials comparing PBI with WBI in breast cancer patients treated with breast-conserving therapy. Four studies including 2,185 patients were selected. Compared to WBI, the pooled odds ratios (OR) for contralateral breast cancer were 0.86 (95% confidence interval (CI) 0.31–2.42; *p* = 0.78) after 5 years and 1.15 (95% CI 0.43-3.09; *p* = 0.78) after 10 years for PBI. The pooled ORs for secondary non-breast cancer were 0.91 (95% CI 0.49-1.67; *p* = 0.77) after 5 years and 1.20 (95% CI 0.39-3.66; *p* = 0.75) after 10 years for PBI compared to WBI. These results demonstrate that the risk of secondary malignancies is similar for PBI and WBI after breast-conserving radiotherapy.

## INTRODUCTION

Breast cancer is the most common cancer among European and North American women [1]. Clinical data suggest that adjuvant radiotherapy, which decreases loco-regional recurrence rates and increases survival, might play an important role in early breast cancer treatment [2–5]. As an increasing number of women become long-term survivors of breast cancer, more research on radiation-induced secondary cancer is needed [6]. Several studies have reported a positive correlation between radiotherapy for breast cancer and the risk of secondary malignancies [2, 7–9]. Additionally, radiotherapy in normal tissue is still required for long-term survivors of breast cancer, although the absolute risks associated with this treatment are relatively small.

Breast-conserving therapy (BCT), a safe and standard procedure, is currently used in early-stage breast cancer patients. Compared to radical mastectomy alone, BCT in combination with whole breast irradiation (WBI) improves local disease control as well as overall survival [10, 11]. By reducing the irradiation field to the quadrant in which the carcinoma arose, partial breast irradiation (PBI), which involves a larger dose of adjuvant therapy after BCT, provides an alternative to WBI. Trials indicate that PBI is associated with better local control rates and cosmetic outcomes [12, 13].

Additional information about the risk of secondary malignancies associated with different PBI techniques, such as intraoperative radiotherapy (IORT), external-beam radiotherapy, and brachytherapy, would allow for better management of local therapy. However, the rarity of secondary malignancies in early-stage breast cancer patients makes such analyses difficult. We therefore conducted a systematic review and meta-analysis to detect meaningful differences between PBI and WBI with a larger sample size and more statistical power.

## RESULTS

### Literature selection and characteristics

**Figure 1:**
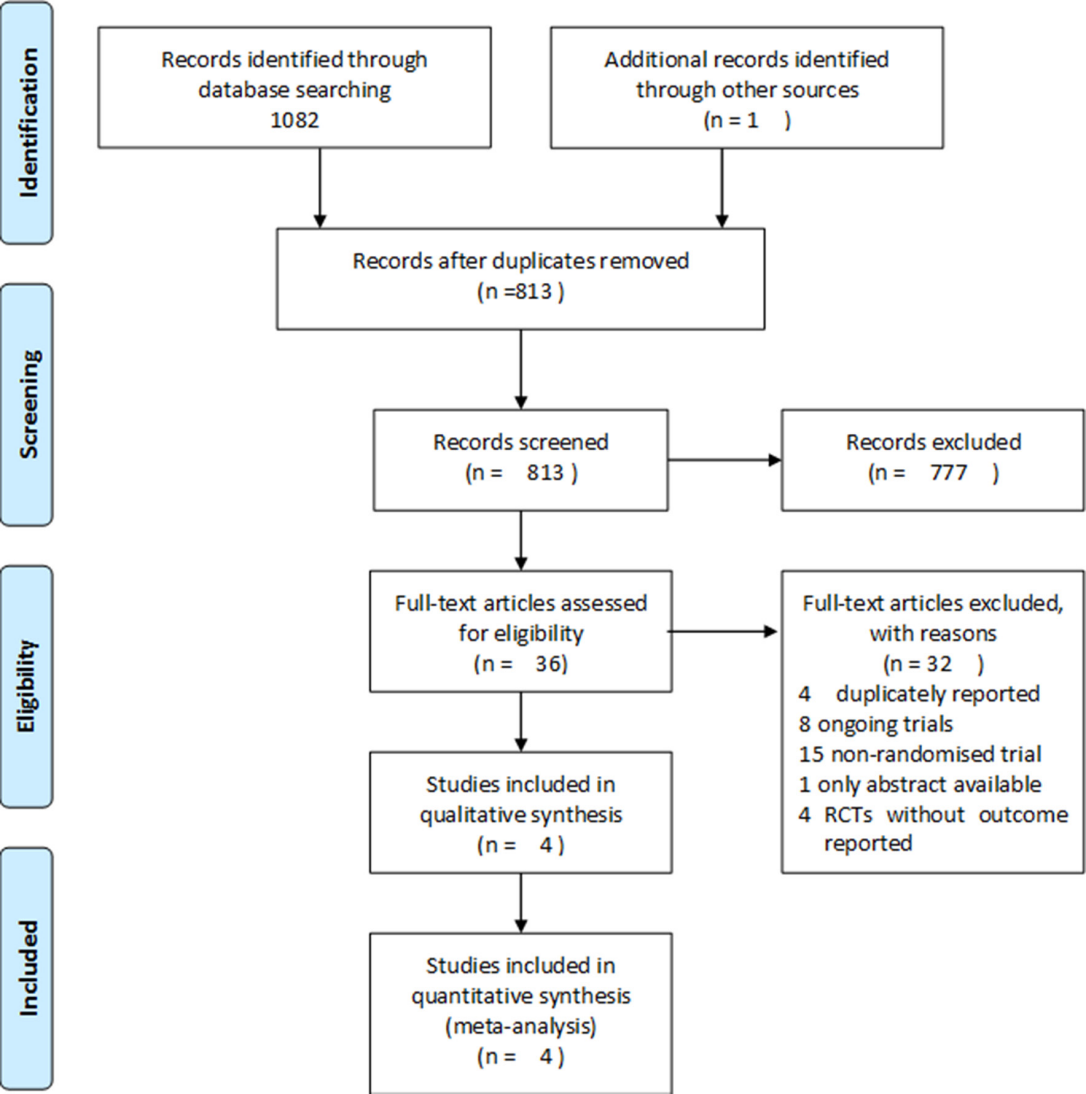
Flowchart depicting the study selection process

The process used to evaluate articles for inclusion is depicted in Figure 1. In total, of the 1,082 titles reviewed, 8 randomized controlled trials [14–21] were identified, and 4 studies [14–17] involving 2,185 patients were ultimately selected. Characteristics of the included studies are summarized in Table 1.

**Table 1:** Characteristics of included studies

Study	Enrollment period	Population			Follow-Up (median)	Chemotherapy		Hormone therapy		Both		Radiotherapy		Surgery	T stage	N stage	Grade 3
		PBI	WBI	Total		PBI	WBI	PBI	WBI	PBI	WBI	PBI	WBI				
Polgar	1998-2004	128	130	258	66 m	2	2	87	89	0	3	HDR MI 36.4 Gy/7fx	42-50 Gy/21-25fx	wide excision,	T1-2	N0:249	I-II
Hungary, 2013												or electrons		negative margins.		N1-2:9	
												42-50 Gy/21-25fx		and/or ALND/SLNB			
ELIOT	2000-2007	651	654	1305	69.6 m	53	47	489	485	84	96	21 Gy/1fx	50 y/25fx +	wide excision	T1-2	N0:949	I-II:989
Italy, 2013												IORT	boost (10 Gy/5fx)	or quadrantectomy		N1:276	III:274
												Electrons		and SLNB or ALND		N2-3:69	
Rodriguez	/	51	51	102	60 m	1	2	50	51	nc	nc	37.5 Gy/10fx	48 Gy/24fx +/−	breast-conserving	T1-2	N0	I-II
Spain, 2013												twice daily	boost (10 Gy/5fx)	surgery			
												3D-CRT					
Livi	2005-2013	260	260	520	60 m	5	3	155	162	7	20	30 Gy/5fx	50 Gy/25fx +	wide excision or	Tis-2	N0:445	I-II:461
Italy, 2015												IMRT	boost (10 Gy/5fx)	quadrantectomy.		N1-3:75	III:59
														clear margins			

Abbreviations: PBI=partial breast irradiation; WBI=whole breast irradiation; HDR MI=High-dose-rate multicatheter interstitial; IMRT=intensity-modulated radiotherapy;

IORT=intraoperative radiotherapy; 3D-CRT=3D conformal external beam radiotherapy; ALND=axillary lymph node dissection; SLNB=sentinel lymph node biopsy.

### Quality assessment

The methodological quality of the included studies was assessed independently by two reviewers and is presented in Table 2. Patients and/or outcome assessors were not blind to experimental conditions, indicating an obvious risk of bias in the included studies. Nevertheless, two studies [15, 17] had a low risk of bias; risk of bias was unclear for the other two studies [14, 16].

**Table 2:** Methodology quality assessment

Items	Polgar	ELIOT	Rodriguez	Livi
1. Random sequence generation	√	√	√	√
2. Allocation concealment	√	√	√	√
3. Blinding of participants and personnel	×	×	×	×
4. Blinding of outcome assessment	×	×	×	×
5. Incomplete outcome data addressed	√	√	√	√
6. Selective reporting	?	√	√	√
7. Free of vested interest bias	√	√	√	√
8. Free of baseline imbalance	√	√	√	√
9. Free of early stopping bias	×	√	√	√
10. Free of expertise bias	?	√	√	√

Note: √ = yes; × = no; ? = not clear

### Contralateral breast cancer

**Figure 2:**
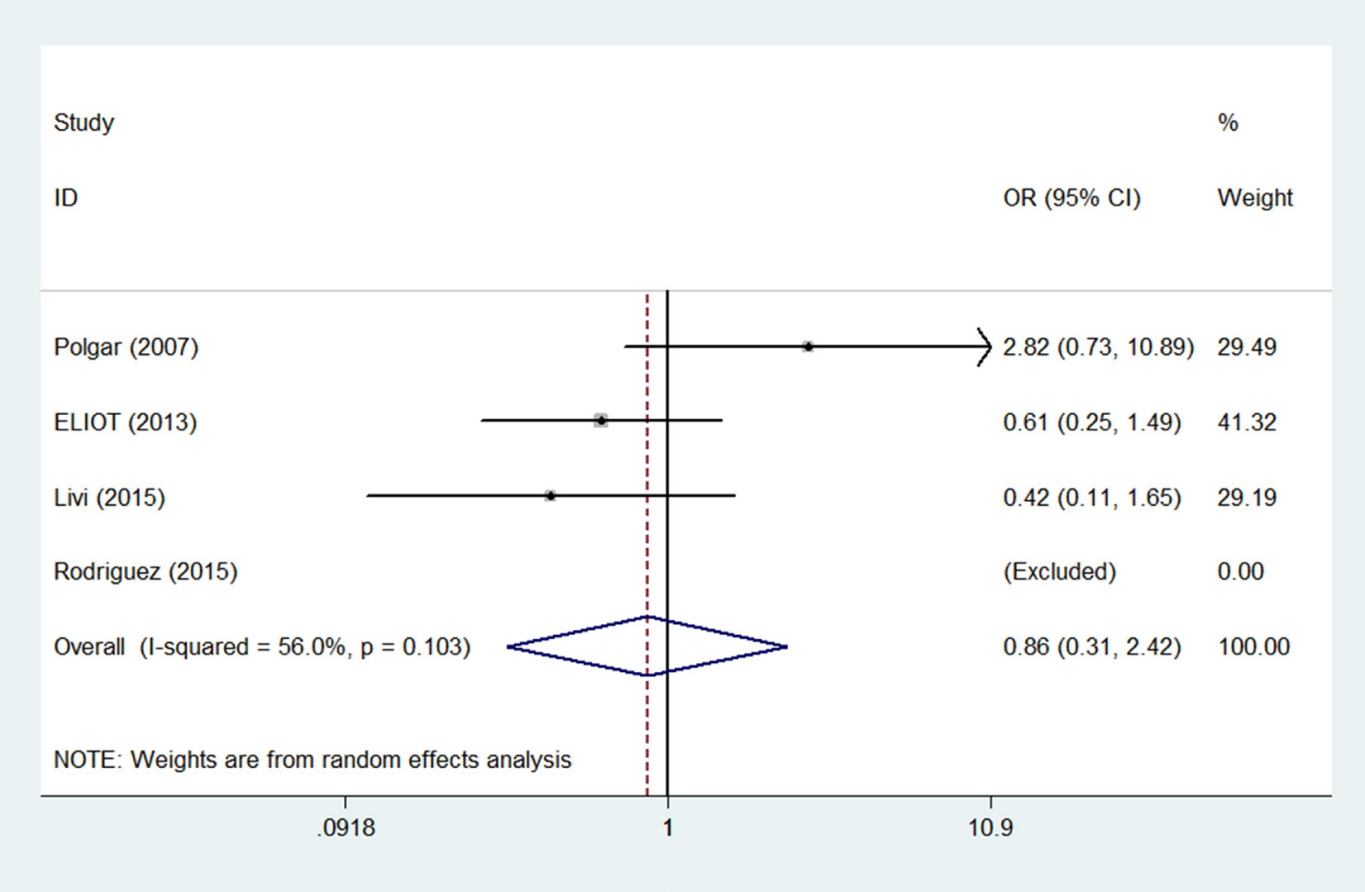
Pooled odds ratios for contralateral breast cancer within 5 years of partial versus whole breast irradiation

**Figure 3:**
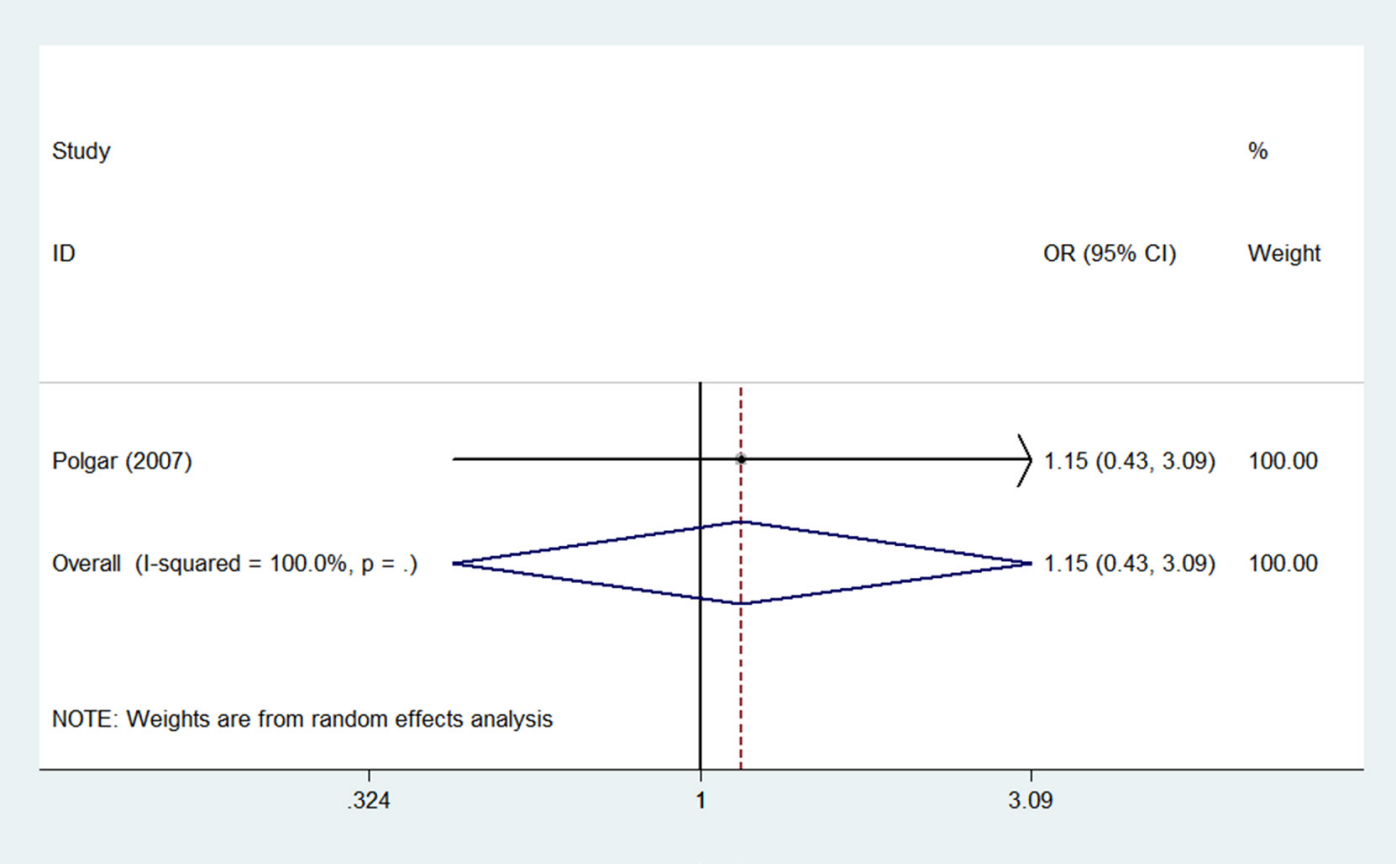
Pooled odds ratios for contralateral breast cancer within 10 years of partial versus whole breast irradiation

All four studies reported contralateral breast cancer occurrences within 5 years, and one study [14] also reported 10-year contralateral breast cancer outcomes. Compared to WBI, the pooled OR of contralateral breast cancer for PBI was 0.86 (95% confidence interval (CI) 0.31–2.42; *p* = 0.78) after 5 years (Figure 2) and 1.15 (95% CI 0.43–309; *p* = 0.78) after 10 years (Figure 3). Meta-analysis revealed a similar risk of contralateral breast cancer between the PBI and WBI groups, with significant heterogeneity across the four trials (*p*(Q) = 0.13, I ^2^ = 56.0%).

### Secondary non-breast cancer

**Figure 4:**
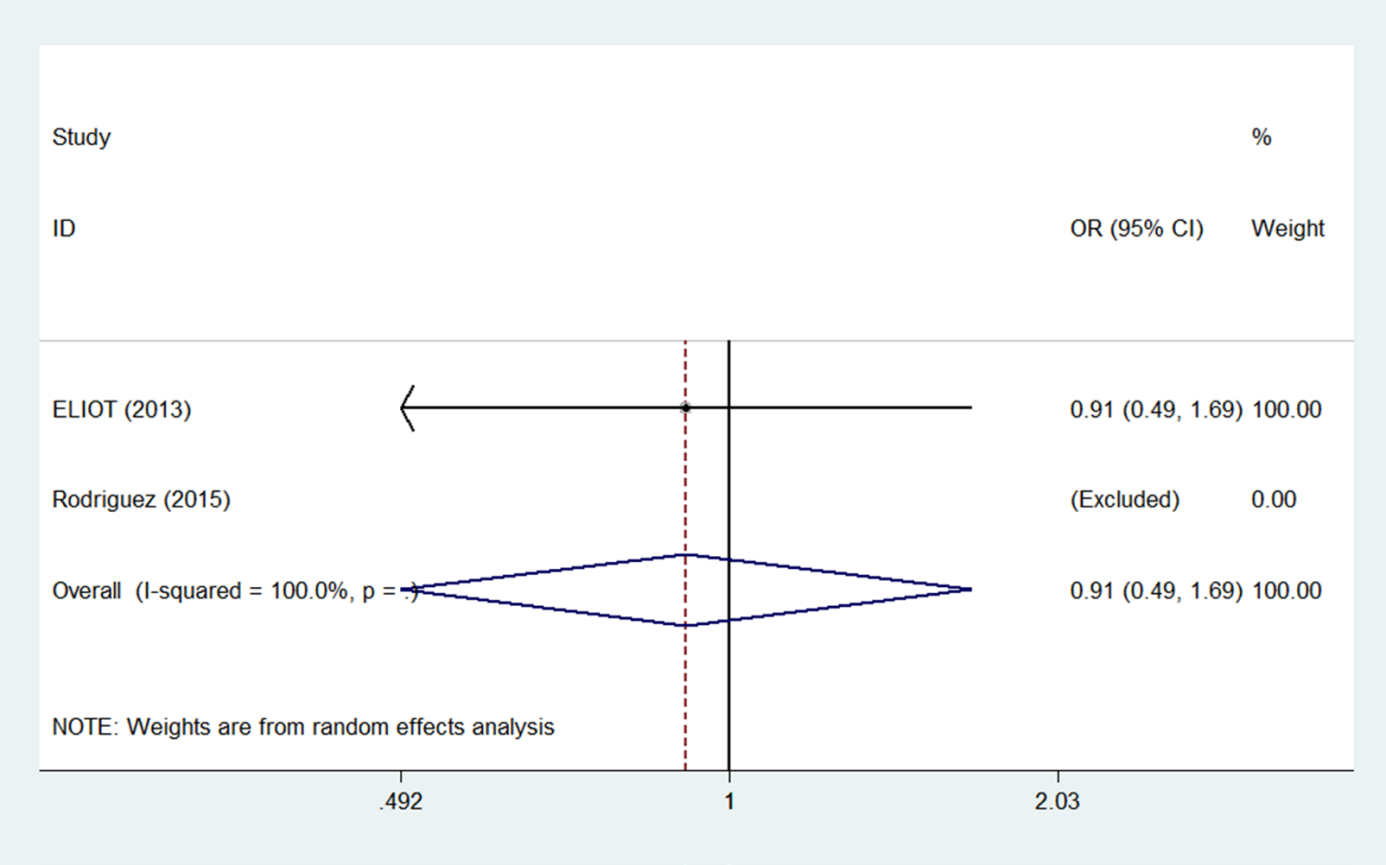
Pooled odds ratios for secondary non-breast cancer within 5 years of partial versus whole breast irradiation

**Figure 5:**
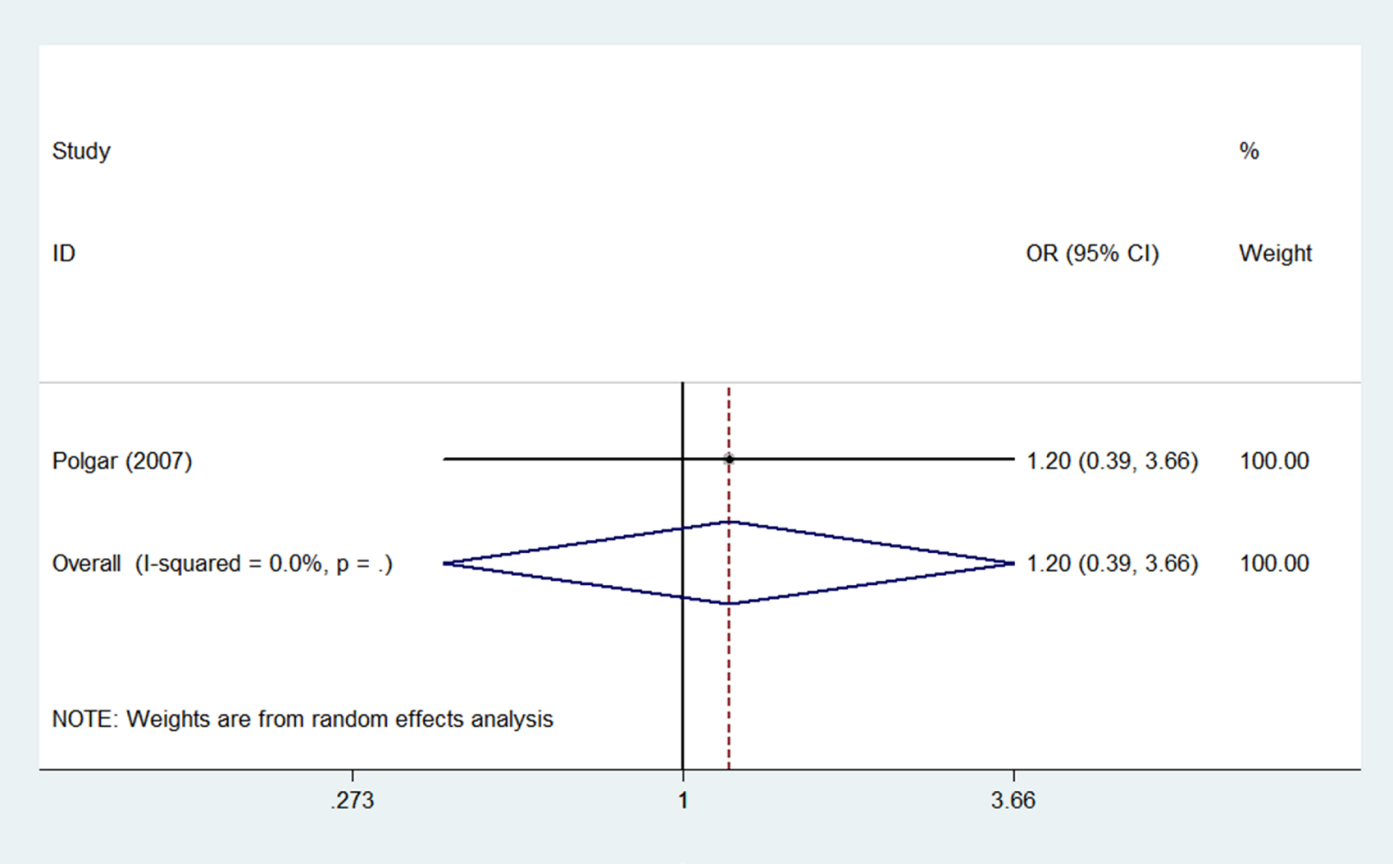
Pooled odds ratios for secondary non-breast cancer within 10 years of partial versus whole breast irradiation

Two studies [15, 16] reported secondary non-breast cancers within 5 years, and one study [14] also reported 10-year secondary non-breast cancer outcomes. The pooled OR of secondary non-breast cancer was 0.91 (95% CI 0.49–1.67; *p* = 0.77) after 5 years (Figure 4) and 1.20 (95% CI 0.39– 3.66*; p* = 0.75) after 10 years (Figure 5) for PBI compared to WBI. Meta-analysis revealed a similar risk of secondary non-breast cancers between the PBI and WBI groups.

### Impact of latency

Risks of contralateral breast cancer (four studies [14–17], OR = 0.86, 95% CI 0.31–2.42; *p* = 0.78) and secondary non-breast cancer (two studies [15, 16], OR = 0.91, 95% CI 0.49–1.67; *p* = 0.77) were similar between PBI- and WBI-treated patients at the 5-year follow-up. Similar results were obtained when analysis was restricted to the study with the 10-year follow-up [14]; the ORs were 1.20 (95% CI 0.39–3.66; *p* = 0.75) for secondary non-breast cancer and 1.15 (95% CI 0.43–3.09; *p* = 0.78) for contralateral breast cancer.

### Impact of treatment techniques

One of the studies examined [15] reported rates of contralateral breast cancer and secondary non-breast cancer in patients treated by IORT with 21 Gy/1fx. In the second study [16], patients received PBI by 3D-RCT with 37.5 Gy/10fx. In the third study [17], patients received IMRT with 30 Gy/5fx as the PBI treatment. In the final study [14], PBI was administered using interstitial high-dose-rate (HDR) implants with 36.4 Gy/7fx or electron beams with 42–50 Gy/21-25fx. The risk of secondary malignancies compared to WBI was similar for these different PBI techniques.

## DISCUSSION

Over the last decade, a growing number of new PBI techniques, such as IORT and IMRT, have been applied in clinical practice. In the ongoing effort to improve treatments and disease control rates while reducing treatment-related morbidity, PBI is becoming an increasingly popular radiotherapy method for breast cancer. However, associations between the risk of secondary malignancies and these novel treatment methods need to be evaluated further over longer follow-up periods. Until now, theoretical models have been used to predict the risk of secondary malignancies when these treatments are used. Although it has better dose conformity and target coverage than WBI, PBI can also be administered using 3D-CRT, which has higher out-of-beam doses [22]. This is important because decreased doses in cancer-adjacent tissues is associated with a decreased risk of secondary cancers in other organs. Thus, the risk of secondary cancer may be higher with 3D-CRT than with WBI. Compared to IMRT, the risk of secondary cancer after 3D-CRT was reduced by 34% in a linear model and 50% in linear-exponential and plateau models [23]. This might be a result of low-level radiation exposure in normal tissues, which in turn resulted from the combined effects of the larger number of treatment fields used in IMRT and radiation leakage produced by these modulated fields. Our meta-analysis found no significant differences in risk of secondary malignancies between 3D-CRT/IMRT and WBI for early breast cancer. However, the small number of studies and patients included here may have limited our ability to detect such differences. Moreover, the median follow-up time (5 years) was relatively short, and the 3D-CRT/IMRT techniques used for PBI in the studies examined remain experimental.

IORT, a novel treatment technique, has increasingly been used after BCT to treat early breast cancer. Compared to WBI, IORT reduces peak doses in nearby normal tissues, the size of the irradiation field, and the treatment time [24]. Although higher local recurrence rates were observed in 5-year follow-ups after ELIOT and TARGIT-A treatments [15, 18], the absolute risk of local recurrence after IORT is less than that in patients undergoing WBI [2, 25]. Additionally, the risk of secondary non-breast and contralateral breast cancer after ELIOT was similar to that observed with PBI and WBI. The risk of secondary malignancies after TARGIT-A treatment was not reported, but the results suggested that IORT reduced mortality due to secondary malignancies compared to WBI [18]. Early breast cancer patients might therefore benefit from IORT treatment rather than WBI, especially when the time period required for WBI and limited access to radiotherapy centers are considered. Additionally, IORT might help a large number of women suitable for BCT to avoid undergoing quadrantectomies or mastectomies [26–28].

The significant heterogeneity among the four studies examined might result from differences among the WBI groups and the various radiation types administered to the PBI groups. All WBI treatments were delivered using a conventional isocentric tangential two-field technique with a dose of 42–50 Gy/21-25fx. Two studies [15, 17] also administered a boost dose of 10 Gy/5fx after WBI. One study [14] did not deliver a boost dose, while the last study [16] delivered an additional 10 Gy/5fx to the tumor bed only in some cases depending on risk factors for local recurrence. Radiation of regional lymph nodes in the WBI groups also differed among the four studies. Heterogeneous radiotherapy techniques were also used in the PBI groups. The PBI group was treated with 36.4 Gy/7fx using multi-catheter interstitial high-dose-rate or with 42-50 Gy/21-25fx by electron beam using 6–15 MeV enface electron fields to the tumor bed and extended to a margin of 2 cm in Polgar *et al*. [14]. In ELIOT [15], the electron intraoperative radiotherapy technique was conducted using two dedicated linear accelerators, and all patients received one full dose of 21 Gy to the tumor bed after tumor removal. Patients received 37.5 Gy/10fx twice daily, separated by at least 6 hours, for 5 consecutive working days via 3D-CRT in Rodriguez *et al*. [16]. In Livi *et al*., a dose of 30 Gy/5fx non-consecutive daily fractions was administered via IMRT. Although the differences in PBI techniques might have contributed to heterogeneity among the studies, the risk of contralateral breast cancer was similar in all studies. This indicated that the pooled OR was stable and reliable.

Four previous meta-analyses have examined the efficiency of PBI compared to WBI for early breast cancer treatment [13, 29–31]. However, only one of these studies assessed the risk of contralateral breast cancer after PBI compared to WBI [30]. Additionally, that meta-analysis included only three randomized trial studies with 5-year follow-ups, a comparative study with a 7-year follow-up, and a matched-pair analysis with a 10-year follow-up. There were no differences in 5-year (OR = 2.82 (95% CI 0.73–10.89, *p* = 0.13)), 7-year (OR = 0.19 (95% CI 0.01–4.00, *p* = 0.28)), or 10-year (OR = 0.48 (95% CI 0.20–1.15, *p* = 0.10)) risk of contralateral breast cancer between PBI and WBI. However, the authors of that study pointed out that the results should be interpreted with caution due to the inclusion of only one randomized controlled trial. We obtained similar results in this meta-analysis, in which we examined 4 RCTs involving a total of 2,185 non-treated breast cancer patients. Contralateral breast cancer rates were similarly low following PBI and WBI; furthermore, no treatment-dependent differences were found after a longer follow-up period. However, none of the studies examined here compared secondary non-breast cancer risks after PBI and WBI.

The following limitations of the present systematic review should be considered when interpreting the results. First, there was a significant risk of bias because researchers and/or outcome assessors were not blind to patient treatments. However, it is unlikely that observations were influenced by the absence of independent assessment, which is not necessarily crucial in this type of intervention. Second, only 4 studies were included in our systematic review, and the risk of secondary malignancies was very low in all of them, perhaps reducing the statistical power of our analysis. In addition, differences among the WBI groups and the specific types of radiation delivered to the PBI groups might contribute to significant heterogeneity among the four studies. Finally, none of the included studies provided information on potential confounding variables which might affect secondary cancer rates, such as smoking status, alcohol consumption, body mass index, and genetic factors. Of particular note is the lack of information on chemotherapy for both the PBI and WBI groups; additionally, the relationship between chemotherapy and risk of secondary malignancies was not examined in the studies.

In conclusion, the risk of secondary malignancies after is similar after PBI and WBI treatment in breast cancer. Compared to rates of other complications associated with breast cancer treatment, the absolute rates of these secondary cancers were quite low. These findings may be helpful for guiding decisions regarding treatment for breast cancer patients.

## MATERIALS AND METHODS

### Study criteria

All randomized controlled trials were eligible; quasi-randomized and non-randomized studies were excluded. Systemic treatments were allowed.

### Participants

Patients in eligible studies were diagnosed with breast cancer by histopathology and treated with BCT and radiotherapy.

### Interventions and controls

PBI treatments were delivered using 3D conformal external beam radiotherapy (3D-CRT), intensity-modulated radiation therapy (IMRT), IORT, or interstitial/intracavitary implants. WBI treatments with or without boost radiotherapy served as controls.

### Outcome measures

Contralateral breast cancer was the primary outcome, and secondary non-breast cancer was the secondary outcome.

### Search methods

The MEDLINE, EMBASE, and Cochrane Library databases were searched from inception through December 1, 2015 by two independent investigators without any language restrictions. Searches were conducted using a combination of the free terms ‘breast cancer,’ ‘breast conservation therapy,’ ‘whole breast irradiation,’ and ‘partial breast irradiation.’ To identify additional relevant studies, the references of the initially identified research and review papers were examined as well.

### Study selection

Randomized trials were retrieved and assessed to by two independent reviewers. Any controversies were resolved by a third reviewer.

### Quality assessment

The risk of bias in the included studies was estimated using the tool recommended by the Cochrane collaboration.

### Statistical analysis

Pooled estimates are presented as odds ratios (OR) with 95% confidence intervals (95%CI). The Cochran Q test and I^2^ statistics were employed to assess heterogeneity among the studies. For the *Q* test, *p* < 0.05 indicated the presence of heterogeneity; for I^2^ statistics, I^2^ > 50% indicated severe heterogeneity. A random-effects model was chosen if significant heterogeneity existed (*p* < 0.05 or I^2^ > 50%); otherwise, a fixed-effects model was adopted. Sensitivity analysis was also conducted to further investigate the sources of heterogeneity. A funnel plot was used to assess publication bias. All statistical analyses were performed using STATA version 12.0 (STATA, College Station, TX, USA).

## References

[1] Forouzanfar MH, Foreman KJ, Delossantos AM, Lozano R, Lopez AD, Murray CJ, Naghavi M (2011). Breast and cervical cancer in 187 countries between 1980 and 2010: a systematic analysis. Lancet.

[2] Clarke M, Collins R, Darby S, Davies C, Elphinstone P, Evans V, Godwin J, Gray R, Hicks C, James S, MacKinnon E, McGale P, McHugh T (2005). Effects of radiotherapy and of differences in the extent of surgery for early breast cancer on local recurrence and 15-year survival: an overview of the randomised trials. Lancet.

[3] Darby S, McGale P, Correa C, Taylor C, Arriagada R, Clarke M, Cutter D, Davies C, Ewertz M, Godwin J, Gray R, Pierce L, Whelan T (2011). Effect of radiotherapy after breast-conserving surgery on 10-year recurrence and 15-year breast cancer death: meta-analysis of individual patient data for 10,801 women in 17 randomised trials. Lancet.

[4] Overgaard M, Jensen MB, Overgaard J, Hansen PS, Rose C, Andersson M, Kamby C, Kjaer M, Gadeberg CC, Rasmussen BB, Blichert-Toft M, Mouridsen HT (1999). Postoperative radiotherapy in high-risk postmenopausal breast-cancer patients given adjuvant tamoxifen: Danish Breast Cancer Cooperative Group DBCG 82c randomised trial. Lancet.

[5] Overgaard M, Hansen PS, Overgaard J, Rose C, Andersson M, Bach F, Kjaer M, Gadeberg CC, Mouridsen HT, Jensen MB, Zedeler K (1997). Postoperative radiotherapy in high-risk premenopausal women with breast cancer who receive adjuvant chemotherapy. Danish Breast Cancer Cooperative Group 82b Trial. N Engl J Med.

[6] Tubiana M (2009). Can we reduce the incidence of second primary malignancies occurring after radiotherapy? A critical review. Radiother Oncol.

[7] Grantzau T, Mellemkjaer L, Overgaard J (2013). Second primary cancers after adjuvant radiotherapy in early breast cancer patients: a national population based study under the Danish Breast Cancer Cooperative Group (DBCG). Radiother Oncol.

[8] Berrington de Gonzalez A, Curtis RE, Gilbert E, Berg CD, Smith SA, Stovall M, Ron E (2010). Second solid cancers after radiotherapy for breast cancer in SEER cancer registries. Br J Cancer.

[9] Grantzau T, Overgaard J (2015). Risk of second non-breast cancer after radiotherapy for breast cancer: a systematic review and meta-analysis of 762,468 patients. Radiother Oncol.

[10] Jacobson JA, Danforth DN, Cowan KH, d'Angelo T, Steinberg SM, Pierce L, Lippman ME, Lichter AS, Glatstein E, Okunieff P (1995). Ten-year results of a comparison of conservation with mastectomy in the treatment of stage I and II breast cancer. N Engl J Med.

[11] Fisher B, Anderson S, Bryant J, Margolese RG, Deutsch M, Fisher ER, Jeong JH, Wolmark N (2002). Twenty-year follow-up of a randomized trial comparing total mastectomy, lumpectomy, and lumpectomy plus irradiation for the treatment of invasive breast cancer. N Engl J Med.

[12] Offersen BV, Overgaard M, Kroman N, Overgaard J (2009). Accelerated partial breast irradiation as part of breast conserving therapy of early breast carcinoma: a systematic review. Radiother Oncol.

[13] Marta GN, Macedo CR, Carvalho Hde A, Hanna SA, da Silva JL, Riera R (2015). Accelerated partial irradiation for breast cancer: systematic review and meta-analysis of 8653 women in eight randomized trials. Radiother Oncol.

[14] Polgar C, Fodor J, Major T, Sulyok Z, Kasler M (2013). Breast-conserving therapy with partial or whole breast irradiation: ten-year results of the Budapest randomized trial. Radiother Oncol.

[15] Veronesi U, Orecchia R, Maisonneuve P, Viale G, Rotmensz N, Sangalli C, Luini A, Veronesi P, Galimberti V, Zurrida S, Leonardi MC, Lazzari R, Cattani F (2013). Intraoperative radiotherapy versus external radiotherapy for early breast cancer (ELIOT): a randomised controlled equivalence trial. Lancet Oncol.

[16] Rodriguez N, Sanz X, Dengra J, Foro P, Membrive I, Reig A, Quera J, Fernandez-Velilla E, Pera O, Lio J, Lozano J, Algara M (2013). Five-year outcomes, cosmesis, and toxicity with 3-dimensional conformal external beam radiation therapy to deliver accelerated partial breast irradiation. Int J Radiat Oncol Biol Phys.

[17] Livi L, Meattini I, Marrazzo L, Simontacchi G, Pallotta S, Saieva C, Paiar F, Scotti V, De Luca Cardillo C, Bastiani P, Orzalesi L, Casella D, Sanchez L (2015). Accelerated partial breast irradiation using intensity-modulated radiotherapy versus whole breast irradiation: 5-year survival analysis of a phase 3 randomised controlled trial. Eur J Cancer.

[18] Vaidya JS, Wenz F, Bulsara M, Tobias JS, Joseph DJ, Keshtgar M, Flyger HL, Massarut S, Alvarado M, Saunders C, Eiermann W, Metaxas M, Sperk E (2014). Risk-adapted targeted intraoperative radiotherapy versus whole-breast radiotherapy for breast cancer: 5-year results for local control and overall survival from the TARGIT-A randomised trial. Lancet.

[19] Ribeiro GG, Magee B, Swindell R, Harris M, Banerjee SS (1993). The Christie Hospital breast conservation trial: an update at 8 years from inception. Clin Oncol (R Coll Radiol).

[20] Dodwell DJ, Dyker K, Brown J, Hawkins K, Cohen D, Stead M, Ash D (2005). A randomised study of whole-breast vs tumour-bed irradiation after local excision and axillary dissection for early breast cancer. Clin Oncol (R Coll Radiol).

[21] Olivotto IA, Whelan TJ, Parpia S, Kim DH, Berrang T, Truong PT, Kong I, Cochrane B, Nichol A, Roy I, Germain I, Akra M, Reed M (2013). Interim cosmetic and toxicity results from RAPID: a randomized trial of accelerated partial breast irradiation using three-dimensional conformal external beam radiation therapy. J Clin Oncol.

[22] Joosten A, Matzinger O, Jeanneret-Sozzi W, Bochud F, Moeckli R (2013). Evaluation of organ-specific peripheral doses after 2-dimensional, 3-dimensional and hybrid intensity modulated radiation therapy for breast cancer based on Monte Carlo and convolution/superposition algorithms: implications for secondary cancer risk assessment. Radiother Oncol.

[23] Abo-Madyan Y, Aziz MH, Aly MM, Schneider F, Sperk E, Clausen S, Giordano FA, Herskind C, Steil V, Wenz F, Glatting G (2014). Second cancer risk after 3D-CRT, IMRT and VMAT for breast cancer. Radiother Oncol.

[24] Aziz MH, Schneider F, Clausen S, Blank E, Herskind C, Afzal M, Wenz F (2011). Can the risk of secondary cancer induction after breast conserving therapy be reduced using intraoperative radiotherapy (IORT) with low-energy x-rays?. Radiat Oncol.

[25] Polgar C, Fodor J, Major T, Nemeth G, Lovey K, Orosz Z, Sulyok Z, Takacsi-Nagy Z, Kasler M (2007). Breast-conserving treatment with partial or whole breast irradiation for low-risk invasive breast carcinoma-5-year results of a randomized trial. Int J Radiat Oncol Biol Phys.

[26] Voti L, Richardson LC, Reis I, Fleming LE, Mackinnon J, Coebergh JW (2006). The effect of race/ethnicity and insurance in the administration of standard therapy for local breast cancer in Florida. Breast Cancer Res Treat.

[27] Nattinger AB, Kneusel RT, Hoffmann RG, Gilligan MA (2001). Relationship of distance from a radiotherapy facility and initial breast cancer treatment. J Natl Cancer Inst.

[28] Lazovich DA, White E, Thomas DB, Moe RE (1991). Underutilization of breast-conserving surgery and radiation therapy among women with stage I or II breast cancer. JAMA.

[29] Valachis A, Mauri D, Polyzos NP, Mavroudis D, Georgoulias V, Casazza G (2010). Partial breast irradiation or whole breast radiotherapy for early breast cancer: a meta-analysis of randomized controlled trials. Breast J.

[30] Ye XP, Bao S, Guo LY, Wang XH, Ma YP, Zhang W, Wang CH, Zhang YF, Zhi F, Gao Y, Tian JH, Li R, Gao HM (2013). Accelerated partial breast irradiation for breast cancer: a meta-analysis. Transl Oncol.

[31] Zhang L, Zhou Z, Mei X, Yang Z, Ma J, Chen X, Wang J, Liu G, Yu X, Guo X (2015). Intraoperative Radiotherapy Versus Whole-Breast External Beam Radiotherapy in Early-Stage Breast Cancer: A Systematic Review and Meta-Analysis. Medicine (Baltimore).

